# Natural Image Reconstruction from fMRI Based on Node–Edge Interaction and Multi–Scale Constraint

**DOI:** 10.3390/brainsci14030234

**Published:** 2024-02-28

**Authors:** Mei Kuang, Zongyi Zhan, Shaobing Gao

**Affiliations:** College of Computer Science, Sichuan University, Chengdu 610065, China; qiang_meii@163.com (M.K.); gao_shaobing@163.com (S.G.)

**Keywords:** brain decoding, natural image reconstruction, fMRI, node–edge interaction, multi–scale constraint

## Abstract

Reconstructing natural stimulus images using functional magnetic resonance imaging (fMRI) is one of the most challenging problems in brain decoding and is also the crucial component of a brain–computer interface. Previous methods cannot fully exploit the information about interactions among brain regions. In this paper, we propose a natural image reconstruction method based on node–edge interaction and a multi–scale constraint. Inspired by the extensive information interactions in the brain, a novel graph neural network block with node–edge interaction (NEI–GNN block) is presented, which can adequately model the information exchange between brain areas via alternatively updating the nodes and edges. Additionally, to enhance the quality of reconstructed images in terms of both global structure and local detail, we employ a multi–stage reconstruction network that restricts the reconstructed images in a coarse–to–fine manner across multiple scales. Qualitative experiments on the generic object decoding (GOD) dataset demonstrate that the reconstructed images contain accurate structural information and rich texture details. Furthermore, the proposed method surpasses the existing state–of–the–art methods in terms of accuracy in the commonly used n–way evaluation. Our approach achieves 82.00%, 59.40%, 45.20% in n–way mean squared error (MSE) evaluation and 83.50%, 61.80%, 46.00% in n–way structural similarity index measure (SSIM) evaluation, respectively. Our experiments reveal the importance of information interaction among brain areas and also demonstrate the potential for developing visual–decoding brain–computer interfaces.

## 1. Introduction

Understanding the neural mechanisms of the human visual system is a hot topic in neuroscience. When the visual system receives external stimuli, the human brain encodes the information and produces specific neural responses. Human visual decoding aims to establish the mapping from the given brain activity to the input stimulus information [1,2]. Functional magnetic resonance imaging (fMRI) indirectly reflects the neuron population response by measuring the local variation in the blood oxygen level. Due to the non–invasive and high–spatial–resolution properties, fMRI is widely used in human visual decoding [1,2]. According to the task, human visual decoding research can be divided into three categories: semantic classification [3], image recognition [4], and image reconstruction [2]. The aim of semantic classification is to predict the stimulus category from brain activity. Image recognition requires the model to identify the seen image from a set of candidate stimuli. Due to the complexity and the low signal–to–noise ratio (SNR) property of the fMRI signal, image reconstruction is the most challenging task, intending to reconstruct the entire stimulus from the brain activity pattern. Developing a natural image reconstruction model can theoretically help us to understand how the brain encodes stimulus information, while practically exploring potential solutions for brain–computer interfaces.

Traditional image reconstruction methods rely on machine learning tools such as linear regression [5,6], Bayesian modeling [7,8], and principle component analysis [9] to estimate the pixel values or hand–crafted features from the fMRI. These methods can decode simple stimulus images, such as letters and numbers. However, when applied to complex natural scenes, these algorithms often fail to produce faithful reconstructions due to the simplicity of the models.

With the rapid development of deep neural networks, deep–learning–based image reconstruction methods have been proposed [2,10,11,12,13,14,15,16,17,18,19,20,21,22,23,24,25]. These approaches are classified into two categories: approximation–based approaches [10,11,12,13,14,15,16,17,18] and generation–based approaches [19,20,21,22,23,24,25]. Approximation–based approaches aim to improve the pixel–level similarity between the stimulus and the reconstruction via training well–designed networks from scratch, while generation–based approaches aim to improve the semantic–level consistency by utilizing the powerful pre–trained generative models such as generative adversarial network (GAN), variational autoencoder (VAE), and diffusion model (DM).

Although significant progress has been made in deep–learning–based image reconstruction methods, there is still room for improvement in several aspects. Firstly, the input fMRI signals originate from different brain regions, and there are extensive information interactions between these areas in the underlying mechanisms of the visual system. However, previous studies merely consider the fMRI signals from different regions as a whole high–dimensional vector input of the reconstruction model [11,12,13,14,15,16,17,18,19,20,21,22,23,24,25], which neglects the nature of information exchange that should be considered during the processing. As a result, the limited extracted features lead to poor performance of the model. Secondly, the information contained within an image at multiple scales has a powerful expressive capability. Lower–resolution scales provide the global image structure and higher–resolution scales describe the fine image details. Previous single–scale approaches are insufficient for fully exploiting image information [10,11,12,13,14,15,16,17,19,20,21,22,23,24,25], resulting in blurry reconstructed images under the situation of limited training samples.

**Figure 1:**
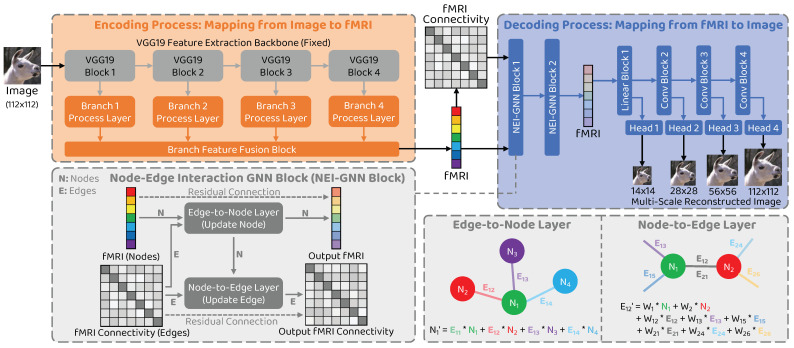
Overview of the proposed approach. The proposed framework comprises two processes: the encoding process and the decoding process. The graph neural network block with node–edge interaction (NEI–GNN block) with edge–to–node and node–to–edge layers models the interaction among brain regions via alternatively updating nodes and edges in the graph. The final reconstructed image is produced by the multi–scale reconstruction network.

Driven by the preceding limitation analysis, we propose a novel natural image reconstruction approach to recover the seen image from the given fMRI brain activity that incorporates node–edge interaction in the graph network to fully model the interactions between different brain regions and introduces multi–scale constraint to improve the reconstructed image quality. Our approach is shown in Figure 1. Following the encoder–decoder methods [11,12,13], two types of model are trained: an encoding model $M_{enc}$ to map from stimulus image to corresponding fMRI activity and a decoding model $M_{dec}$ to map from fMRI signal to the seen image. To overcome the limitation of neglecting information interactions, we consider individual brain regions as nodes and connectivity between them as edges in the graph and develop a novel graph neural network block with node–edge interaction (NEI–GNN block) to model the interactions. Additionally, a multi–scale decoding model is employed to restrict the reconstructed image at various scales, thereby promoting global structure similarity and local detail consistency simultaneously.

Our contributions are as follows: (1) We propose a novel natural image reconstruction approach to recover the seen image from the given fMRI brain activity that incorporates node–edge interaction in the graph network to fully model the interactions between different brain regions and introduces multi–scale constraint to improve the reconstructed image quality in terms of global structure and local detail. (2) To overcome the limitation of neglecting information interactions in the previous study, we consider individual brain regions as nodes and connectivity between them as edges in the graph and develop a novel NEI–GNN block to model the interactions. By stacking multiple NEI–GNN blocks, the high–order long–range dependencies can be captured without introducing additional computational overhead in a single NEI–GNN block. (3) Additionally, a multi–scale decoding model is employed to restrict the reconstructed image at various scales, thereby promoting global structure similarity and local detail consistency simultaneously in a coarse–to–fine manner. (4) Extensive qualitative and quantitative experiments on a publicly available dataset demonstrate the superiority of the proposed methods in terms of visual inspection and objective assessments. In the subjective inspection, the reconstructions obtained by our model successfully retain accurate contours and produce abundant texture details. In the objective evaluation, our method surpasses the existing state–of–the–art methods in the commonly used n–way evaluation metrics.

## 2. Related Work

### 2.1. Natural Image Reconstruction Methods Based on Deep Learning

#### 2.1.1. Approximation–Based Methods

Approximation–based methods often involve designing the effective network and training the model from scratch, without relying on excessive pre–trained components. The characteristic of these methods is high pixel–level similarity. Three types of implementations can be divided: (1) Iterative optimization. Shen et al. [10] estimated the reconstructed image by continuously iterating the image with the objective of minimizing the distance between the features of the reconstructed image and the features decoded from fMRI. (2) Autoencoder. Beliy et al. [11] utilized the encoder–decoder structure to integrate self–supervised learning (SSL) into the decoding model. The encoder learns the mapping from image to fMRI, while the decoder learns the mapping from fMRI to image. By stacking the encoder and decoder, the model can be trained in an SSL manner using unlabeled data. Gaziv et al. [12] and Qiao et al. [13] further improved this method via multi–level feature similarity loss and alternative encoder–decoder regularization, respectively. (3) Generative adversarial network. Seeliger et al. [14] employed the DCGAN as the generator and trained a conversion network to transfer the fMRI activity to the input latent variable of the generator. Shen et al. [15] achieved end–to–end image reconstruction training using image, feature, and adversarial loss. Fang et al. [16] proposed Shape–Semantic GAN, with a shape decoder to decode the shape from low–level visual areas and a semantic decoder to decode the category from high–level visual regions. The outputs from the shape and semantic decoders were used as inputs to an image generation network to reconstruct the stimulus. Ren et al. [17] utilized a dual–path VAE–GAN network structure and trained the model using the knowledge distillation paradigm. Meng et al. [18] adopted a hierarchical network for image feature extraction and reconstruction, combining an fMRI decoder to produce intermediate features for the network to obtain faithful reconstructions.

#### 2.1.2. Generation–Based Methods

Generation–based methods often exploit powerful pre–trained generative models, combine relatively simple fMRI decoders to convert the brain activity to the input or intermediate variables of the generative models, or obtain reconstructions with high semantic–level consistency via the strong generation ability. Two types of implementations can be divided: (1) Generative adversarial network. Mozafari et al. [19] trained a vanilla linear regression model as an fMRI decoder to learn the mapping from fMRI to the input latent variables of the pre–trained BigBiGAN [26]. Reconstructions with high fidelity were obtained by combining the above components. Ozcelik et al. [20] further enhanced this model with another powerful model called ICGAN [27]. Lin et al. [21] utilized the pre–trained CLIP model [28] to extract image and text features from the stimulus image and corresponding caption. An fMRI decoder comprising convolutional operations and linear layers was employed to align the fMRI activity to the CLIP feature space via contrastive learning. Finally, a pre–trained StyleGAN2 [29] was adopted to achieve stimulus image reconstruction. (2) Diffusion model. Chen et al. [22] trained an fMRI feature extraction model using the masked signal modeling learning paradigm [30] and used the fMRI features as the conditional inputs to fine–tune the pre–trained latent diffusion model (LDM) [31]. Ni et al. [23] optimized the implementation of masked signal modeling to further improve the image quality. Meng et al. [24] developed a vanilla linear network to map the fMRI signal to the features extracted by the pre–trained CLIP model [28]. The decoded features were then combined with the reverse process of LDM [31] to produce reconstructions. Lu et al. [25] adopted an LDM [31] to obtain initial reconstruction and then iteratively updated the input variables with the objective of structural similarity between the reconstruction and the corresponding groundtruth.

### 2.2. Graph Neural Network

A graph is a kind of data structure consisting of objects (nodes) and the relationships between them (edges). It is used to represent data in non–Euclidean spaces such as social networks, knowledge dependencies, and so on. Graph neural network (GNN) is a deep learning model that extracts features from the topological information of graphs via information exchange between the nodes and has shown promising results in a variety of node–, edge– and graph–level tasks [32]. In fMRI activity, brain regions and the functional connectivity between them exhibit an explicit graph structure. Therefore, some researchers have attempted to incorporate GNN into the processing model. Kawahara et al. [33] proposed BrainNetCNN for neurodevelopment prediction using novel edge–to–edge, edge–to–node, and node–to–graph layers to capture the topological relationship between brain areas. Li et al. [34] further advanced this approach by introducing multi–order path information aggregation. Meng et al. [35] developed a visual stimulus category decoding model based on the graph convolutional neural network, which is used to extract the functional correlation features between different brain regions. Saeidi et al. [36] employed a graph neural network to decode task–fMRI data, combining a graph convolutional operation and various node embedding algorithms. However, previous approaches based on graph neural network have focused solely on either nodes or edges, neglecting the importance of interactions between them, which restricts the expressive power of the model. In our work, the limitation mentioned above has been taken into consideration, resulting in the proposed novel NEI–GNN block, which highlights the interactions between nodes and edges.

### 2.3. Multi–Scale Constraint

In modern convolutional neural networks, the feature extraction process is typically divided into several stages to extract multi–scale features from images [37]. Features with high resolution contain fine details, while features with low resolution provide coarse image structure. By adding multi–scale constraint to the network, the model can effectively utilize the information at different levels, resulting in improved performance and robustness. For instance, SSD [38] advanced the object detection system performance by predicting on feature maps of various scales simultaneously. DeepLabV3+ [39] boosted the semantic segmentation accuracy by integrating local and global embedding through the atrous convolution and multi–scale feature fusion module. In the field of natural image reconstruction from brain activity, Miyawaki et al. [5] reconstructed the arbitrary binary contrast patterns by separately predicting on predefined multi–scale local image bases. Luo et al. [40] proposed DA–HLGN–MSFF, which combines the hierarchical feature extraction and multi–scale feature fusion block to improve the reconstruction performance. Meng et al. [18] exploited a similar multi–scale encoder–decoder architecture to achieve promising natural image reconstruction. Inspired by Miyawaki et al. [5], our work introduces multi–scale constraint to natural image reconstruction and establishes the joint optimization between scales, instead of a separate restraint on each scale, as in [5].

## 3. Materials and Methods

### 3.1. Dataset

In this study, the experiment was conducted on the generic object decoding (GOD) dataset [3], which is used to fully evaluate the visual image reconstruction performance. The GOD dataset consists of stimulus images from ImageNet [41] and the corresponding fMRI recordings of five subjects. The fMRI SNR values for the five subjects are 0.0649, 0.0613, 0.1045, 0.0924, and 0.0654. It should be noted that the categories of the 1200 training images do not overlap with the categories of the 50 testing images. Each training image was presented once, while each testing image was presented 35 times. To obtain multi–resolution stimulus images, the original 112 × 112 images were downsampled using cubic interpolation. Seven regions of interest (ROI) were used, containing V1, V2, V3, V4, LOC, FFA, and PPA, each with a varying number of voxels. The V1–V4 are identified via the standard retinotopy experiment, and the LOC, FFA, and PPA are delineated using the functional localizers that capture the voxels with significantly higher activations to objects, faces, or scenes than the scrambled images. The voxel numbers for each ROI of the five subjects are as follows: Subject 1—1004, 1018, 759, 740, 540, 568, and 356; Subject 2—757, 944, 810, 544, 834, 435, and 316; Subject 3—872, 1031, 861, 754, 996, 928, and 496; Subject 4—719, 855, 929, 704, 668, 725, and 398; Subject 5—659, 891, 907, 860, 566, 929, and 550. To eliminate the dimension discrepancy, the fMRI vectors of different ROIs were padded with values close to zero to align them. Refer to the original paper of GOD [3] for more details.

### 3.2. Model Overview

The overall structure of the proposed network is illustrated in Figure 1. We denote *I* and *F* to represent the image data and the corresponding fMRI data, respectively. Our framework comprises two processes: the encoding process $M_{enc}$, which maps a stimulus image to the corresponding fMRI activity (I→F), and the decoding process $M_{dec}$, which reconstructs the entire seen image from the given brain signal (F→I). The aim of developing the encoding model is to promote the decoding model by stacking them together and training them in a self–supervised manner using unlabeled image data. To address the issue of ignoring the interactions between brain areas, we introduce a novel NEI–GNN block to model the information exchange between the brain regions, considering brain regions as nodes and their connectivity as edges in the graph. Furthermore, the decoding model consists of multiple stages to produce multi–scale reconstructed images. We restrict the reconstructions at various scales to promote the reconstruction performance in a coarse–to–fine fashion.

In the training phase, the input stimulus image is first transferred to the estimated fMRI response via the encoding model, which is described as follows:

(1) $$F_{e}=M_{enc}\left(I_{r}\right)$$

where $I_{r}$ and $F_{e}$ denote the stimulus image and the estimated corresponding fMRI signal, respectively.

Next, the estimated fMRI activity is separated according to the ROIs, and the initial fMRI connectivity matrix is obtained by taking the dot–product of the fMRI signal from different ROIs, which is described as follows:

(2) $$C_{e}^{i,j}=\frac{F_{e}^{i}\cdot F_{e}^{j}}{∥F_{e}^{i}{∥}_{2}\;*\;{∥F_{e}^{j}∥}_{2}}$$

where $C_{e}^{i,j}$ denotes the estimated fMRI connectivity between ROI *i* and ROI *j*, $F_{e}^{r}$ denotes the fMRI activity from a specified ROI *r*, and r∈{V1,V2,V3,V4,LOC,FFA,PPA}.

Then, the estimated fMRI activity and connectivity are fed into the NEI–GNN blocks, allowing full information exchange by integrating information from adjacent nodes, which outputs the fMRI signals after interaction. The above process can be described as follows:

(3) $$F_{e}^{\prime }=M_{NEI-GNN}\left(F_{e},C_{e}\right)$$

Finally, the output multi–scale reconstructed images are acquired via prediction heads of the reconstruction model at different stages, which is described as follows:

(4) $$I_{e}^{14},I_{e}^{28},I_{e}^{56},I_{e}^{112}=M_{recon}\left(F_{e}^{\prime }\right)$$

where $I_{e}^{14}$, $I_{e}^{28}$, $I_{e}^{56}$, and $I_{e}^{112}$ denote the reconstructed image with 14 × 14, 28 × 28, 56 × 56, and 112 × 112 spatial resolution, respectively.

In the testing phase, the input fMRI activity is processed according to Equations (2) to (4), and the output with 112 × 112 resolution is regarded as the final reconstructed image of the proposed method.

### 3.3. Network Structure: Encoder

The structure of the encoder network in the proposed approach is identical to that in the previous study [12]. A pre–trained VGG19 model is employed as the backbone for image feature extraction, without updating its parameters during the training process. The features from the layers relu1_2, relu2_2, relu3_4, and relu4_4 are regarded as multi–branch features. The features of each branch then pass through the branch–specific processing layer, which includes a spatial feature processing module and a channel feature processing module. The output fMRI estimation is obtained by fusing the multi–branch outcomes through the branch feature fusion block. Please refer to the original paper of GazivEncDec [12] for further details.

### 3.4. Network Structure: Decoder

The decoder comprises two NEI–GNN blocks, along with the multi–stage reconstruction network. Further information on the NEI–GNN block is provided in the following section. To enhance the quality of reconstructed images, we propose the multi–stage reconstruction network that constrains the image in global structure and local detail. This approach differs from the use of multiple models to predict stimulus images at different scales separately [5], as we uniformly constrain multiple scales in a single model. This is achieved by the reconstruction backbone network with multiple scale–specific reconstruction heads. Please refer to Table 1 for the detailed network configuration.

### 3.5. Network Structure: Node–Edge Interaction GNN (NEI–GNN) Block

The visual system’s underlying brain mechanisms involve numerous brain area information interactions that enable various brain regions to collaborate and achieve complex visual system functions. For instance, the processing of edge information is supported by the interaction between V1 and V2 [42]. The dorsal pathway extensively interacts with the ventral pathway [43]. The above descriptions illustrate the numerous interactions of information in the brain. Functional connectivity is an expression of the interaction between brain regions, indicating the degree of similarity of signals between them [44]. Previous studies have shown that decoding can be accomplished based on either brain area signals [5] or connectivity signals [45], indicating that both types of signals contain effective features. Thus, by combining signals from different brain regions and their connectivity in a specific manner, more comprehensive features can be extracted.

To model the brain area information interaction behaviour, we consider individual brain regions as nodes and the connections between them as edges in the graph, respectively. The NEI–GNN block takes into account not only the nodes and edges in the graph but also the interactions between them. Figure 1 shows the schematic diagram of the module, which is divided into an edge–to–node layer and a node–to–edge layer. The functions of each layer are described below in detail.

In the edge–to–node layer, each node value after interaction is obtained by aggregating the values of itself and its directly adjacent nodes, using the edges as weights. This is calculated using the following equation:

(5) $$N_{i}^{\prime }=\sum_{j=1}^{R}E_{i,j}\;*\;N_{j}$$

where $N_{r}$ denotes the vector of the fMRI from a specified ROI *r* and $E_{i,j}$ is a scalar and denotes the connectivity between the ROI *i* and *j*.

In the node–to–edge layer, each edge value after interaction is computed by collecting the values of itself, its directly related nodes, and their edges. Learnable parameters are used to fuse this information, and the calculation is performed using the following equation:

(6) $$E_{i,j}^{\prime }=w_{i}\cdot N_{i}^{\prime }+w_{j}\cdot N_{j}^{\prime }+\sum_{k_{1}=1}^{R}w_{i,k_{1}}\;*\;E_{i,k_{1}}+\sum_{k_{2}=1}^{R}w_{j,k_{2}}\;*\;E_{j,k_{2}}$$

where $w_{r}$ denotes the learnable vector used to weight the voxel activations in $N_{r}$, and $w_{r_{1},r_{2}}$ denotes the learnable scalar used to weight the connectivity between ROI $r_{1}$ and $r_{2}$. The first two terms compute the contribution of the vertexes by calculating the dot–product of the learnable vector and the corresponding node data, and the following two terms determine the contribution of the adjacent edges.

**Figure 2:**
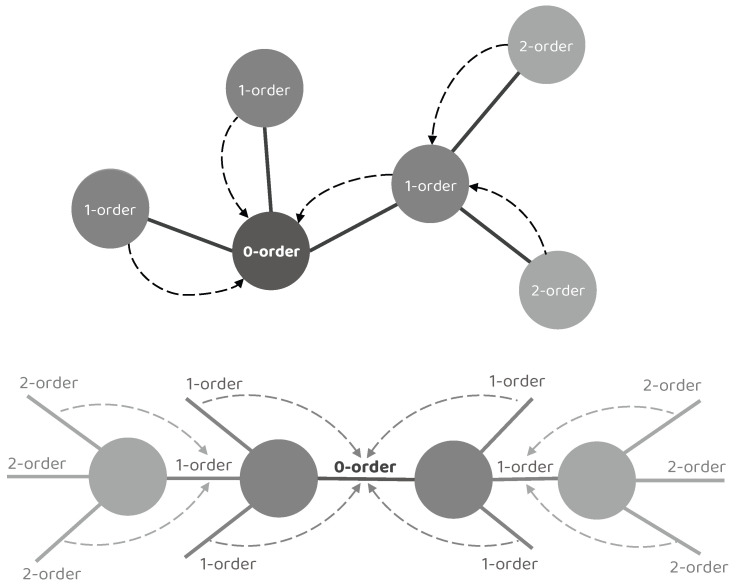
Illustration of the high–order interaction modeling by stacking multiple NEI–GNN blocks. A single NEI–GNN block only gathers the information from adjacent nodes or edges, resulting in limited information utilization of merely 0–order and 1–order collection. By stacking multiple NEI–GNN blocks together, the high–order node/edge information could first be aggregated by the 1–order node/edge in the previous NEI–GNN block, then the high–order long–range dependencies can be captured by the following NEI–GNN blocks.

Concretely, we denote the target node/edge to be updated as a 0–order node/edge, its adjacent node/edge as a 1–order node/edge, the adjacent node/edge of the 1–order node/edge as a 2–order node/edge, and so on. It is worth noting that a single NEI–GNN block only gathers the information from adjacent nodes or edges, resulting in limited information utilization of merely 0–order and 1–order collection. However, numerous indirect connections also exhibit significant impact. By stacking multiple NEI–GNN blocks together, the high–order long–range dependencies can be captured without introducing additional computational overhead in a single NEI–GNN block, as the high–order node/edge information could be aggregated by the 1–order node/edge in the previous NEI–GNN block. Figure 2 illustrates the aforementioned concept.

### 3.6. Training Strategy

**Figure 3:**
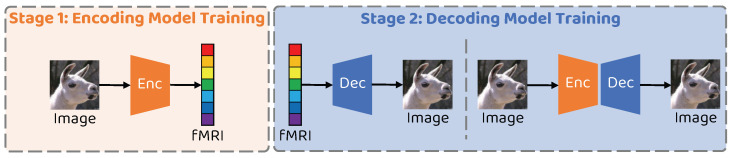
Schematic illustration of the two–stage training strategy of the proposed method.

The training process of the proposed network is mainly divided into two phases, the encoder training phase and the decoder training phase, as illustrated in Figure 3. All experiments in this paper are conducted using an Intel 12700KF CPU and an NVIDIA RTX3080 GPU with 12GB memory. The proposed network is implemented using PyTorch 1.7.1, with an overall training time of approximately 3 h.

#### 3.6.1. Training Stage 1: Encoder Training

During the encoder training stage, supervised training is used with image–fMRI paired data. The stimulus images serve as the encoder input, and the corresponding fMRI records are used as the training labels. The loss function consists of the mean squared error loss and the cosine similarity loss [46], as shown below:

(7) $$L_{enc}=L_{F-MSE}+L_{F-CosSim}$$

(8) $$L_{F-MSE}=\frac{1}{N}\sum_{i=1}^{N}{\left(F_{e}^{\left(i\right)}-F_{r}^{\left(i\right)}\right)}^{2}$$

(9) $$L_{F-CosSim}=-\frac{F_{e}\cdot F_{r}}{∥F_{e}{∥}_{2}\;*\;{∥F_{r}∥}_{2}}$$

where N denotes the number of elements in the fMRI data and $F_{e}$ and $F_{r}$ denote the estimated and groundtruth fMRI data, respectively.

#### 3.6.2. Training Stage 2: Decoder Training

During the decoder training phase, the encoder’s parameters remain fixed and are not updated. The training objectives consist of two parts: (1) Supervised training using paired fMRI–image data. The fMRI signals are used as inputs to the decoder, while the corresponding images are used as labels. (2) Self–supervised training using unlabeled image data by stacking the encoder and decoder to form an autoencoder architecture. The loss function of the above two parts consists of the mean absolute error loss, the perceptual loss [47], and the total variation loss [48], as illustrated below:

(10) $$L_{dec}=L_{SL}+L_{SSL}$$

(11) $$L_{SL}=L_{SSL}=L_{I-MAE}+L_{I-Per}+L_{I-TV}$$

(12) $$L_{I-MAE}=\frac{1}{HW}\sum_{i=1}^{H}\sum_{j=1}^{W}|I_{e}^{\left(i,j\right)}-I_{r}^{\left(i,j\right)}|$$

(13) $$L_{I-Per}=-\frac{1}{HWL}\sum_{l=1}^{L}\sum_{i=1}^{H}\sum_{j=1}^{W}\frac{\phi^{\left(l,i,j\right)}\left(I_{e}\right)\cdot \phi^{\left(l,i,j\right)}\left(I_{r}\right)}{∥\phi^{\left(l,i,j\right)}\left(I_{e}\right){∥}_{2}\;*\;{∥\phi^{\left(l,i,j\right)}\left(I_{r}\right)∥}_{2}}$$

(14) $$L_{I-TV}=\sum_{i=1}^{H-1}\sum_{j=1}^{W-1}\sqrt{{\left(I_{e}^{\left(i,j+1\right)}-I_{e}^{\left(i,j\right)}\right)}^{2}+{\left(I_{e}^{\left(i+1,j\right)}-I_{e}^{\left(i,j\right)}\right)}^{2}}$$

where *H* and *W* denote the height and width, respectively, of the images or feature maps, *L* denotes the total number of layers used to calculate the perceptual loss, ϕ denotes the fixed VGG16 feature extraction network, and $I_{e}$ and $I_{r}$ denote the estimated and groundtruth image data, respectively. Note that the perceptual loss is only applied to restrict the 112 × 112 scale, while the other losses are applied to all scales.

### 3.7. Evaluation Metric

We use the n–way metric to fully evaluate the performance of the model, which is commonly used in the field of natural image reconstruction from fMRI activity [2]. For each target test stimulus image, N–1 different stimulus images are randomly selected together to form a set of N candidate images. The similarity function between the reconstructed image and each candidate image is then calculated. If the similarity value between the reconstructed image and the target test groundtruth is equal to or even more optimal than any similarity values between the reconstructed image and other candidate images, this test stimulus image is regarded as passing the evaluation. The pass rate across all test stimulus images reflects the performance of the reconstruction approach. In our experiment, the values of N are 2, 5, and 10. Mean squared error (MSE) and structural similarity index measure (SSIM) are used as the similarity function. Aiming to reduce the influence of random selection during the evaluation process, the n–way evaluation is repeated 10 times and the average pass rate is denoted as the final “n–way accuracy”.

In addition to the comparison–based n–way evaluation metrics described above, we also employ image–level metrics to evaluate the reconstruction performance in terms of low–level similarity and high–level consistency. Firstly, the raw MSE and SSIM are utilized to measure the low–level similarity between the stimulus and the reconstructions. Secondly, to fully evaluate the performance, following the previous study [49], we employ the pre–trained CLIP model [28] as the high–level feature extractor and measure the high–level consistency by calculating the distance in the CLIP feature space.

## 4. Results

In this section, we verify the effectiveness of the proposed approach through extensive qualitative and quantitative experiments on the GOD dataset. Firstly, the proposed method is compared with a series of state–of–the–art natural image reconstruction methods in terms of visual inspection and objective assessments. In the following, the ablation experiment results related to the NEI–GNN block and multi–scale constraint are illustrated. Finally, we conduct some analysis experiments to demonstrate the effect of some factors on our method, such as image scale, visual area, and subject. The experiments in this study are mainly conducted on the fMRI data from Subject 3, except for the comparison experiments among different subjects.

### 4.1. Qualitative and Quantitative Comparison Results

#### 4.1.1. Qualitative Comparison

**Figure 4:**
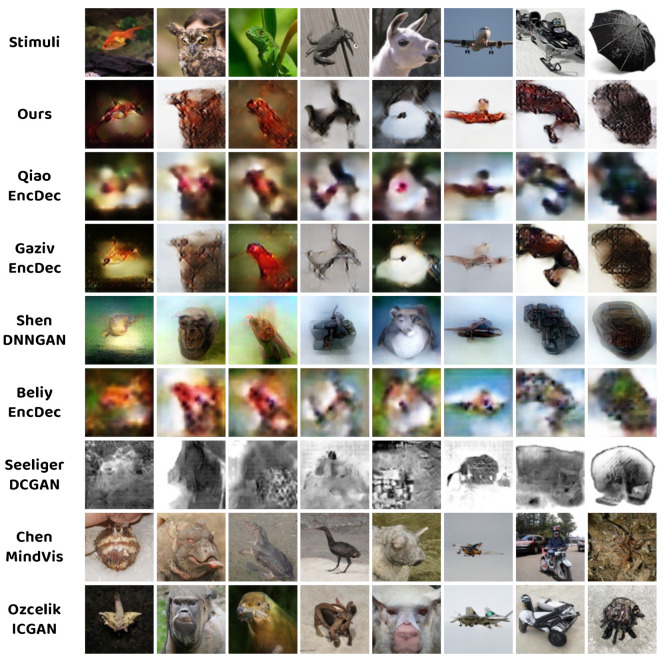
Qualitative comparison between the proposed method and several state–of–the–art methods (Qiao et al., 2022 [13], Gaziv et al., 2022 [12], Shen et al., 2019 [10], Beliy et al., 2019 [11], Seeliger et al., 2018 [14], Chen et al., 2023 [22], and Ozecelik et al., 2022 [20]).

Figure 4 presents the comparison between the proposed method and seven existing state–of–the–art approaches, including five approximation–based methods (QiaoEncDec [13], GazivEncDec [12], ShenDNNGAN [10], BeliyEncDec [11], and SeeligerDCGAN [14]) and two generation–based methods (ChenMindVis [22] and OzecelikICGAN [20]). As demonstrated in Figure 4, our approach successfully achieves natural image reconstruction from the fMRI activity and exhibits accurate contour similarity and superior texture consistency compared to other approximation–based methods. Despite being inferior to generation–based methods in terms of image naturalness, generation–based methods suffer from strong semantic discrepancy in the resulting images.

#### 4.1.2. Quantitative Comparison via N–Way Evaluation Metrics

From an objective perspective, quantitative comparisons are conducted using the n–way identification accuracy mentioned above. The results are illustrated in Table 2. The proposed method achieves promising performance in both MSE n–way accuracy and SSIM n–way accuracy, indicating the effectiveness of the NEI–GNN block and multi–scale constraint in realizing faithful visual stimulus reconstruction.

#### 4.1.3. Quantitative Comparison via Image–Level Metrics

Unlike the comparison–based n–way evaluation metrics, the image–level metrics assess the performance from the perspective of each single image. Table 3 demonstrates the corresponding results. Our method reaches the best results in the raw MSE metric and achieves comparable results in the raw SSIM metric, which exhibits promising performance in terms of low–level similarity. However, due to the approximation–based method nature of the proposed method, the high–level CLIP distance metric obtained by our method still has a gap with that of generation–based methods.

### 4.2. Ablation Study

#### 4.2.1. Effectiveness of the Node–Edge Interaction GNN Block

**Figure 5:**
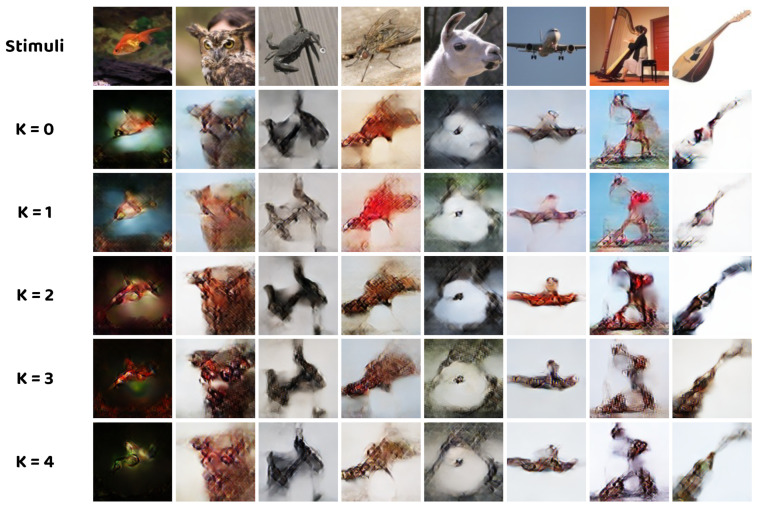
Qualitative comparison between models with different number of NEI–GNN blocks.

Ablation experiments are designed to investigate the role of the proposed novel NEI–GNN block in natural image construction and determine the optimal number of NEI–GNN blocks. As demonstrated in Figure 5, the model with two NEI–GNN blocks produces the best reconstruction results, while the other variants result in more blurry outcomes. It is worth noting that the model without any NEI–GNN blocks outperforms the model with only one NEI–GNN block in certain cases, which can be explained by insufficient modeling of the brain region interaction due to the shallow network. The quantitative results in Table 4 also indicate that K = 2 is the optimal selection for the number of NEI–GNN blocks, which surpasses other variants in n–way accuracies, except for two–way SSIM accuracy, but with comparable metrics. Employing more NEI–GNN blocks degrades the model performance, which can be explained by the over–smooth effect inherent in the deep GNN [50].

#### 4.2.2. Effectiveness of the Multi–Scale Constraint

**Figure 6:**
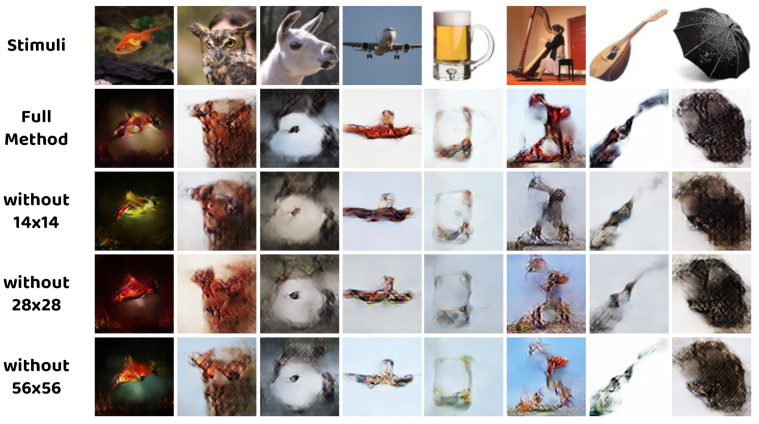
Qualitative comparison between models with different multi–scale constraint.

Similarly, we remove the constraints on the 14 × 14, 28 × 28, and 56 × 56 scale on the basis of the full method. The obtained results are shown in Figure 6. Without the constraints, on all scales, the reconstructed images suffer from many degradations, such as unclear edges, color distortion, and noisy outcomes, which highlights the significance of the multi–scale restrictions considered in the proposed method.

Table 5 presents the corresponding quantitative results of this ablation study. All the leading metrics demonstrate the effectiveness of the multi–scale constraint. Specifically, removing the 56 × 56 scale constraint may not be quite harmful to the performance, while wiping off the restriction on 28 × 28 scale significantly degrades the metrics, which indicates the important role of the intermediate scale.

### 4.3. Effect of Different Scales

**Figure 7:**
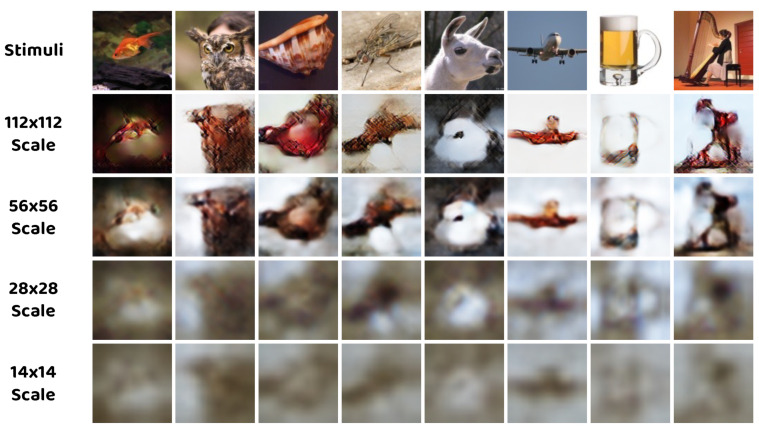
Examples of reconstructed images from different scales of the proposed method.

In order to analyze the effect of different scales on our method, the multi–scale outcomes are visualized in Figure 7. Several patterns could be recognized in the results: Firstly, reconstructions with higher resolution contain rich detail information, whereas low–resolution reconstructions also capture the global structure of the stimuli. Secondly, the 112 × 112 and 56 × 56 scales share similar characteristics of fine details, while the 28 × 28 and 14 × 14 scales share similar characteristics of accurate coarse structure. From Table 5 and Figure 7, we can infer that the reason the performance of the model without the 28 × 28 scale constraint drops significantly is that the 28 × 28 scale provides crucial overall structure guidance to the model and restricts the reconstructions with sharper contours, compared to the 14 × 14 scale with more blurry outputs.

### 4.4. Effect of Different Visual Areas

**Figure 8:**
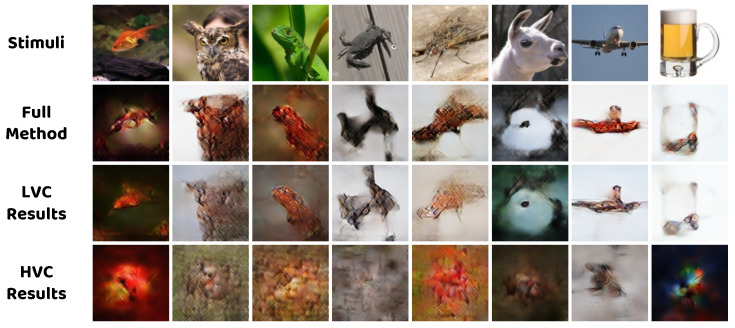
Qualitative comparison between the reconstructed images obtained by models trained using different brain regions.

Here, we demonstrate how different visual areas influence the reconstruction process. Following the previous studies [12,17] which divide the whole visual cortex into subareas, we define the set of brain regions V1, V2, V3, and V4 as the LVC region; the set of brain regions LOC, FFA, and PPA as the HVC region; and the set of all brain regions mentioned above as the VC region. The fMRI activity from LVC, HVC, and VC are used to train the model. Figure 8 and Table 6 illustrate the subjective and objective comparison experiment results. In Figure 8, the model with the LVC region is able to reconstruct most of the structural information but still lacks enough contrast, while no meaningful information could be observed in the reconstructed images from the model with the HVC region. By comparing the results with those of the full method, it could be inferred that the LVC region encodes more structural features, whereas the HVC region encodes more high–level semantic features that effectively improve the image quality. The quantitative results from Table 6 are also in line with the above analysis, with higher metrics from LVC and lower metrics from HVC but topmost metrics from the whole VC.

### 4.5. Effect of Different Subjects

**Figure 9:**
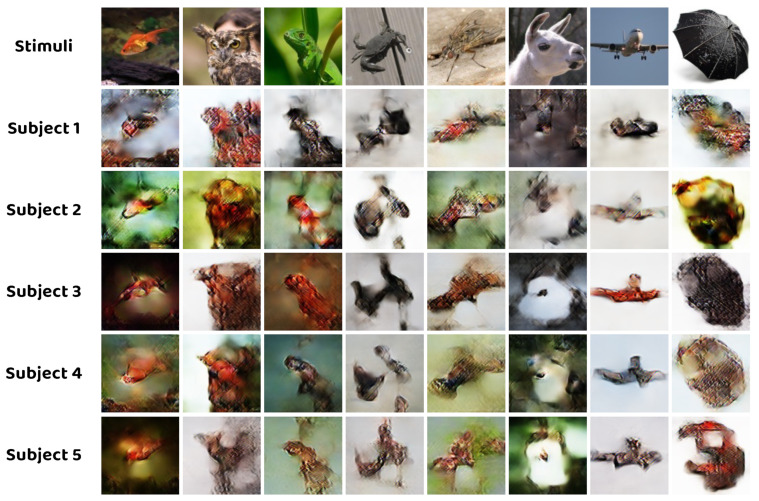
Qualitative comparison between the reconstructed images obtained by models trained using fMRI activity from different subjects.

Ultimately, the effect of different subjects is explored by training the model using fMRI activity from various subjects. The SNR of the fMRI signal is a crucial factor behind the subjects. As observed in Figure 9 and Table 7, individuals with higher SNR produce reconstructions that closely resemble the original stimuli. Conversely, as the SNR decreases, the reconstructed images deteriorate, making it more challenging to identify the predominant object in the image.

## 5. Discussion

In this paper, we proposed a promising natural image reconstruction method to tackle the problem of reconstructing the visual stimuli from fMRI data. The interactions between different brain regions are modeled via the NEI–GNN blocks, considering not only the nodes and edges in the graph but also the interactions between them. Additionally, we exploit the multi–scale constraint to improve the performance in a coarse–to–fine manner. With the help of the above components, our method outperforms several state–of–the–art methods in both qualitative observation and quantitative evaluation.

Extensive experiments illustrate the superiority of the proposed method. In the subjective inspection, the reconstructions obtained by our model successfully retain accurate contours and produce abundant texture details. In the objective evaluation, our method surpasses the existing approaches in terms of MSE n–way accuracy and SSIM n–way accuracy. What makes the presented model outstanding is mainly attributed to the following reasons: Firstly, we employed the NEI–GNN blocks to fully consider the interactions between brain regions, producing more expressive fMRI features that are helpful for the reconstruction. Furthermore, we restrict the reconstructed images in both structural similarity and texture consistency through the multi–scale constraint, resulting in significant performance improvements.

However, due to the approximation–based nature of the proposed method, our method also suffers from lacking enough semantic information and image fidelity. A potential solution to this limitation is introducing the brain region interaction and multi–scale constraint into the generation–based methods, which holds the promise of obtaining promising reconstructed images with not only abundant structural and semantic information but also high image fidelity. For instance, a decoding network with NEI–GNN blocks could be trained to map the fMRI signal to the latent vector of the pre–trained diffusion models, and the latent vector could be fine–tuned to minimize the similarity between the stimulus and the reconstruction in a multi–scale fashion.

As shown in Figure 9, the variability of the reconstructed images obtained using fMRI data from different subjects is another limitation of the methodology in this paper. Future work aims to improve the image quality of reconstructed images in low–SNR scenarios. One potential solution is to construct a noise estimation network to fully characterize the noise in the fMRI signal [51,52]. By removing the noise properly, it can enhance the model’s performance from a data perspective.

Eventually, the current model represents connections between brain regions using adjacency matrices. With the development of the fMRI acquisition, more detailed brain region delineation could be provided. Using adjacency matrices in the implementation is improper as the graph of brain regions is sparse rather than fully connected. Future work could involve exploring a sparse graph representation that is compatible with existing deep learning frameworks. Although this may not enhance the model’s performance, it will certainly improve the method’s practicality.

## 6. Conclusions

A novel natural image reconstruction approach based on node–edge interaction and multi–scale constraint is proposed and demonstrates promising performance in reconstructing the seen image from fMRI data. The proposed method considers individual brain regions as nodes and connectivity between them as edges in the graph. Then, our approach leverages the novel NEI–GNN block to fully model the extensive interactions between brain regions. By stacking multiple NEI–GNN blocks, the high–order long–range dependencies can be well–captured. Additionally, we incorporate the multi–scale constraint into the decoding model to enhance the reconstruction quality in terms of both coarse structure and fine detail. Our method has been demonstrated to be superior through qualitative and quantitative experiments. In the aspect of subjective inspection, the reconstructions of the proposed method present accurate contours and abundant texture details. In the aspect of objective evaluation, our approach surpasses the state–of–the–art methods in the commonly used n–way evaluation process. Despite its strengths, there are still some challenges faced by the proposed model. The lack of enough semantic information and image fidelity should be considered, aiming to produce more realistic reconstructions. In addition, the low–SNR scenarios in the fMRI processing should be taken into account to increase the robustness under the circumstances of acquiring multiple fMRI recordings at different periods or by various subjects. Finally, with the increase in brain regions that could be identified, for the method’s practicality, some compatible improvements should be made, such as sparse graph representation. In conclusion, our method holds the potential to develop promising and practical visual decoding models.

## Figures and Tables

**Table 1:** Network configuration of the multi–stage recontruction network in the decoder.

Module Name	Network Architecture
Linear Block 1	Linear (C = 14 × 14 × 64)
Head 1	Conv2d (K = 5, C = 3), Sigmoid
Conv Block 2	Upsample (×2), Conv2d (K = 5, C = 64), GroupNorm, Swish
Head 2	Conv2d (K = 5, C = 3), Sigmoid
Conv Block 3	Upsample (×2), Conv2d (K = 5, C = 64), GroupNorm, Swish
Head 3	Conv2d (K = 5, C = 3), Sigmoid
Conv Block 4	Upsample (×2), Conv2d (K = 5, C = 64), GroupNorm, Swish
Head 4	Conv2d (K = 5, C = 3), Sigmoid

**Table 2:** Quantitative comparison between the state–of–the–art methods and the proposed method using n–way evaluation metrics. Higher accuracy means better reconstruction performance. The best results are emphasized in bold.

Methods	MSE N–Way Accuracy (%)			SSIM N–Way Accuracy (%)		
	2–Way	5–Way	10–Way	2–Way	5–Way	10–Way
Chance Level (Random Guess)	50.00	20.00	10.00	50.00	20.00	10.00
OzcelikICGAN [20] (IJCNN, 2022)	76.20	44.00	30.20	69.00	36.20	20.40
ChenMindVis [22] (CVPR, 2023)	56.40	25.00	14.40	57.00	27.80	14.60
SeeligerDCGAN [14] (NeuroImage, 2018)	70.20	36.20	23.40	60.00	32.00	16.80
BeliyEncDec [11] (NeurIPS, 2019)	78.00	48.00	34.80	73.80	47.80	34.80
ShenDNNGAN [10] (PLoS Comput Biol, 2019)	76.40	50.60	37.40	70.80	46.20	33.00
GazivEncDec [12] (NeuroImage, 2022)	78.20	55.20	43.80	79.20	58.00	44.00
QiaoEncDec [13] (Biomed Signal Process Control, 2022)	79.40	58.80	44.40	77.80	54.00	41.20
Ours	**82.00**	**59.40**	**45.20**	**83.40**	**61.80**	**46.00**

**Table 3:** Quantitative comparison between the state–of–the–art methods and the proposed method using image–level evaluation metrics. Lower MSE, higher SSIM, and lower CLIP distance mean better reconstruction performance. The best results are emphasized in bold.

Methods	MSE	SSIM	CLIP Distance
OzcelikICGAN [20] (IJCNN, 2022)	0.098	0.161	**0.205**
ChenMindVis [22] (CVPR, 2023)	0.123	0.142	0.216
SeeligerDCGAN [14] (NeuroImage, 2018)	0.102	0.134	0.319
BeliyEncDec [11] (NeurIPS, 2019)	0.101	0.185	0.323
ShenDNNGAN [10] (PLoS Comput Biol, 2019)	0.109	0.195	0.264
GazivEncDec [12] (NeuroImage, 2022)	0.097	**0.225**	0.291
QiaoEncDec [13] (Biomed Signal Process Control, 2022)	0.094	0.195	0.319
Ours	**0.093**	0.215	0.312

**Table 4:** Quantitative comparison between models with different number of NEI–GNN block using n–way evaluation metrics. Higher accuracy means better reconstruction performance. The best results are emphasized in bold.

Model with K NEI–GNN Block	MSE N–Way Accuracy (%)			SSIM N–Way Accuracy (%)		
	2–Way	5–Way	10–Way	2–Way	5–Way	10–Way
K = 0	78.00	56,00	44.40	81.20	60.40	45.20
K = 1	79.00	55.40	42.60	82.00	57.00	40.40
K = 2	**82.00**	**59.40**	**45.20**	83.40	**61.80**	**46.00**
K = 3	79.60	57.00	44.80	**84.00**	61.40	44.20
K = 4	78.40	55.20	39.60	79.60	56.20	37.00

**Table 5:** Quantitative comparison between models with different multi–scale constraint using n–way evaluation metrics. Higher accuracy means better reconstruction performance. The best results are emphasized in bold.

Model	MSE N–Way Accuracy (%)			SSIM N–Way Accuracy (%)		
	2–Way	5–Way	10–Way	2–Way	5–Way	10–Way
w/o 14 × 14 scale constraint	80.80	56.40	43.60	82.40	60.00	42.80
w/o 28 × 28 scale constraint	79.00	52.60	42.40	81.40	58.40	43.40
w/o 56 × 56 scale constraint	81.40	57.20	43.20	82.60	61.00	44.40
Full Method	**82.00**	**59.40**	**45.20**	**83.40**	**61.80**	**46.00**

**Table 6:** Quantitative comparison between the reconstructed images obtained by models trained using different brain regions using n–way evaluation metrics. Higher accuracy means better reconstruction performance. The best results are emphasized in bold.

Model	MSE N–Way Accuracy (%)			SSIM N–Way Accuracy (%)		
	2–Way	5–Way	10–Way	2–Way	5–Way	10–Way
Model with LVC	78.20	51.60	37.20	80.80	56.60	42.20
Model with HVC	65.20	34.80	22.20	57.20	27.00	16.00
Full Method	**82.00**	**59.40**	**45.20**	**83.40**	**61.80**	**46.00**

**Table 7:** Quantitative comparison between the reconstructed images obtained by models trained using fMRI activity from different subjects using n–way evaluation metrics. Higher accuracy means better reconstruction performance. The best results are emphasized in bold.

Model of Different Subject	MSE N–Way Accuracy (%)			SSIM N–Way Accuracy (%)		
	2–Way	5–Way	10–Way	2–Way	5–Way	10–Way
Subject1 (SNR = 0.0649)	70.20	40.00	28.40	73.20	47.80	33.60
Subject2 (SNR = 0.0613)	72.40	47.20	38.20	74.20	50.40	36.20
Subject3 (SNR = 0.1045)	**82.00**	**59.40**	**45.20**	**83.40**	**61.80**	**46.00**
Subject4 (SNR = 0.0924)	74.40	47.40	33.20	77.40	52.20	37.20
Subject5 (SNR = 0.0654)	75.80	49.20	36.00	74.20	50.40	39.80

## Data Availability

The fMRI data used in this research are publicly available at [3].
